# Involvement of the CXCL12 System in the Stimulatory Effects of Prenatal Exposure to High-Fat Diet on Hypothalamic Orexigenic Peptides and Behavior in Offspring

**DOI:** 10.3389/fnbeh.2017.00091

**Published:** 2017-05-17

**Authors:** Kinning Poon, Jessica R. Barson, Huanzhi Shi, Guo Qing Chang, Sarah F. Leibowitz

**Affiliations:** ^1^Laboratory of Behavioral Neurobiology, Rockefeller UniversityNew York, NY, USA; ^2^Department of Neurobiology and Anatomy, Drexel University College of MedicinePhiladelphia, PA, USA

**Keywords:** prenatal high-fat diet, (C-X-C motif) ligand 12 (CXCL12), hypothalamus, anxiety, neuropeptides

## Abstract

Exposure to a high fat diet (HFD) during gestation stimulates neurogenesis and expression of hypothalamic orexigenic neuropeptides that affect consummatory and emotional behaviors. With recent studies showing a HFD to increase inflammation, this report investigated the neuroinflammatory chemokine, CXCL12, and compared the effects of prenatal CXCL12 injection to those of prenatal HFD exposure, first, by testing whether the HFD affects circulating CXCL12 in the dam and the CXCL12 system in the offspring brain, and then by examining whether prenatal exposure to CXCL12 itself mimics the effects of a HFD on hypothalamic neuropeptides and emotional behaviors. Our results showed that prenatal exposure to a HFD significantly increased circulating levels of CXCL12 in the dam, and that daily injections of CXCL12 induced a similar increase in CXCL12 levels as the HFD. In addition, prenatal HFD exposure significantly increased the expression of CXCL12 and its receptors, CXCR4 and CXCR7, in the hypothalamic paraventricular nucleus (PVN) of the offspring. Finally, the results revealed strong similarities in the effects of prenatal HFD and CXCL12 administration, which both stimulated neurogenesis and enkephalin (ENK) expression in the PVN, while having inconsistent or no effect in other regions of the hypothalamus, and also increased anxiety as measured by several behavioral tests. These results focus attention specifically on the CXCL12 chemokine system in the PVN of the offspring as being possibly involved in the stimulatory effects of prenatal HFD exposure on ENK-expressing neurons in the PVN and their associated changes in emotional behavior.

## Introduction

It is currently understood that continued ingestion of a diet rich in fat causes inflammatory responses that lead to chronic disease (Fritsche, 2015; Walker and Harrison, 2015; Welty et al., 2016). In adult animals, ingestion of a high-fat diet (HFD) as well as fatty acid exposure are both found to increase classical inflammatory mediators in the periphery and brain (Milanski et al., 2009; Posey et al., 2009). Further, peripheral or central administration of such inflammatory mediators, in addition to increasing systemic and brain inflammation, can also affect emotional behaviors (Bianchi et al., 1992; Oh et al., 2010), while administration of an inflammatory inhibitor decreases inflammation and consummatory behavior (Posey et al., 2009). Exposure during gestation to inflammatory mediators is also found to have effects on the offspring. In addition to causing increased inflammation in the brain, it alters neuronal function and increases susceptibility to developing obesity (Dahlgren et al., 2001; Gayle et al., 2004; Meyer et al., 2006; Iwasa et al., 2010; Reynolds et al., 2014). Prenatal exposure to a HFD similarly increases endogenous inflammatory mediators in the periphery and brain of the offspring and affects emotional behavior and food intake (Zhu et al., 2008; Stofkova et al., 2009; White et al., 2009; Bilbo and Tsang, 2010; Grayson et al., 2010). While this evidence supports a causal link between HFD exposure and inflammatory processes, it is not clear how and where the immune system when stimulated by a HFD functions to exert its effects on neuronal function during the gestational period and to produce long-lasting changes in the offspring's behavior.

The effects on the brain of HFD exposure during gestation include changes in various neuropeptides in the hypothalamus, which themselves are known to stimulate the ingestion of a HFD (Robert et al., 1989; Gomori et al., 2003; Naleid et al., 2007; Chang et al., 2008), project to brain areas involved in mediating emotional behaviors (Thompson et al., 1996; Kirouac et al., 2006), and have a role in controlling arousal and anxiety-like behaviors (Herman et al., 2003; Chen et al., 2015). In adult rats, a HFD is found to increase the levels and expression of enkephalin (ENK) in the paraventricular nucleus (PVN) and also of orexin (OX) and melanin-concentrating hormone (MCH) in the perifornical lateral hypothalamus (PFLH), while having little effect on or suppressing neuropeptide Y (NPY) in the arcuate nucleus (ARC) (Ikenasio-Thorpe et al., 2007; Chang et al., 2008; Barson et al., 2011). Also, similar effects on these peptide systems, along with an increase in hypothalamic neurogenesis, are observed in the offspring of rats that consumed a HFD while pregnant (Chang et al., 2008). With little known about the molecular and cellular mechanisms involved in mediating these phenomena, we focused our attention on a specific class of inflammatory mediators, called chemokines, which have been shown in several studies to colocalize with and affect neuropeptide expression and function (Banisadr et al., 2002, 2003, 2016; Guyon et al., 2005; Poon et al., 2014). Specifically, the chemokine C-X-C motif chemokine ligand 12 (CXCL12) is found to directly affect ENK expression in the PVN (Poon et al., 2016), influence the firing of MCH neurons in the lateral hypothalamus (Guyon et al., 2005), and play a critical role in the development of the nervous system (Mithal et al., 2012), with genetic knockout of this chemokine causing embryonic fatality (Nagasawa et al., 1996; Ma et al., 1998). Also, the CXCL12 receptors, CXCR4, and CXCR7, are universally expressed throughout the brain, including the hypothalamus, and found to be directly stimulated by CXCL12 to affect neuronal function (Banisadr et al., 2003, 2016), with their targeted deletion shown to induce multiple detrimental changes in physiology and behavior (Sierro et al., 2007; Tsuchiya et al., 2012; Cash-Padgett et al., 2016). Together, this evidence encouraged us to investigate the effects of prenatal exposure to HFD on the CXCL12 chemokine system and determine whether CXCL12 has similar effects to the HFD on the development of specific hypothalamic neuropeptide systems and on the behavior of the offspring.

Thus, to investigate the relation of the CXCL12 chemokine system to the effects of maternal consumption of a HFD on offspring development, this study exposed dams to a HFD or to CXCL12 at two doses and measured changes in circulating CXCL12 in the dam, both CXCL12 and its receptors in offspring brain, and the expression of hypothalamic neuropeptides at two postnatal ages and emotional behaviors in adult offspring. With knockout of the CXCL12 system found to be lethal in embryos and CXCL12 antagonists commercially unavailable, we used CXCL12 itself to manipulate this chemokine system in pregnant rats and compared its effects to those of a HFD on the offspring's brain and behavior, to determine if they have similar actions suggesting their close relationship.

## Methods

### Animals

Timed-pregnant, embryonic day 4 (E4) Sprague-Dawley rats were acquired from Charles River Laboratories (Hartford, CT). All experimental procedures were performed according to institutionally approved protocols as specified in the NIH Guide to the Care and Use of Animals and also with approval of the Rockefeller University Animal Care and Use Committee. The dams were individually housed in a fully accredited AAALAC facility (22°C, with a 12:12-h light-dark cycle with lights off at 12 p.m.) as were all weaned offspring. All efforts were made to minimize the use and suffering of the animals. For the PCR and behavioral tests, 5 groups of dams were used: control, saline injection, HFD, 2 μg CXCL12 and 8 μg CXCL12, with 8 dams per group (*N* = 40). All groups with the exception of the HFD group were maintained *ad libitum* on standard lab chow (3.36 kcal/g) with 13% fat (Purina, St. Louis, MO). To acclimate the HFD dams to the diet, they were given a 15 kcal ball of HFD for 3 days along with the lab chow, after which the lab chow was removed and only the HFD was provided. The HFD access and the daily intraperitoneal injections of saline, 2 or 8 μg CXCL12 began on embryonic day 9 (E9) and continued until E18, when the injections were stopped and the HFD dams were weaned off of the diet over a 3-day period with chow available. This timing corresponds with the critical period of hypothalamic neuronal development (Bouret, 2012). For all groups, the litters were culled to 9 per dam, and the pups stayed with their mother until weaning at postnatal day 21 (P21). At P15, one male offspring from each dam was sacrificed for PCR analysis. Then, one male offspring was sacrificed at P30 for PCR analysis, and at P60, one male from each dam was used for behavioral testing. For the experiments examining the brain using immunofluorescence histochemistry or measuring food intake, diet preference and body weight, another 5 groups of dams (*N* = 40) with 8 dams/group were used: control, saline injection, HFD, 2 μg CXCL12 and 8 μg CXCL12. With one male offspring sacrificed at P15 from each dam in the 5 groups for the immunofluorescence histochemistry analysis (*N* = 25), the remaining offspring (*n* = 8/group) were weaned at P21, and their food intake and body weight were measured every other day. After a 3-day acclimation with a 15 kcal HFD ball from P46–49, a single male at P50 from each dam (*N* = 38) was given a HFD challenge for 7 days, with intake and body weight measured daily. After the acclimation period, the rats were given full access to both the HFD and chow diet over the course of 7 days, and daily measurements of caloric intake and body weight were taken.

### Diet

For the experimental period, unless otherwise stated, rats were maintained *ad libitum* on standard rodent chow (13% fat, 3.3 kcal/g; LabDiet, St. Louis, MO). As described in our previous publications (Dourmashkin et al., 2006; Chang et al., 2008; Poon et al., 2016), the HFD consisted of 50% fat (5.15 kcal/g) from 75% lard (Armour Star, Peoria, IL) and 25% vegetable oil (Crisco, Orrville, OH), 25% carbohydrate from 30% dextrin (ICN Pharmaceuticals, Costa Mesa, CA), 30% cornstarch (ICN Pharmaceuticals, Costa Mesa, CA), and 40% sucrose (Domino Foods Inc., Yonkers, NY), and 25% protein from casein (Bio-Serv, Frenchtown, NJ). It was supplemented with minerals (USP XIV Salt Mixture Briggs; ICN Pharmaceuticals, Costa Mesa, CA) and vitamins (Vitamin Diet Fortification Mixture; ICN Pharmaceuticals, Costa Mesa, CA). This diet, stored at 4°C until use, is nutritionally complete and does not have any detrimental effects on the health of the animals.

### ELISA

Tail vein blood was collected from 5 groups of dams (control, saline injection, HFD, 2 μg CXCL12, 8 μg CXCL12), first at E7 prior to the start of any exposure and then again at E17 to be compared with the initial baseline. Since stress from repeat injections in pregnant dams can have significant behavioral effects (Ryabinin et al., 1999), tail vein blood was collected only twice during the pregnancy. A mouse CXCL12 ELISA kit that is compatible with rat CXCL12 (R&D Systems, Minneapolis, MN) was used to measure serum levels of CXCL12 according to the manufacturer's instructions.

### Brain dissections

The P15 and P30 offspring were sacrificed by rapid decapitation to collect brain tissue for qPCR analysis. Immediately after sacrifice, the brain was placed in a matrix slicing guide with the ventral surface facing up, and four 0.5 mm coronal sections were made, yielding two slices to be used for microdissection. Using the middle optic chiasm as the anterior boundary and the stereotaxic atlas of a 10-day-old rat brain for guidance (Sherwood and Timiras, 1970), a slice was made at the level of A3.8–3.5 mm for microdissection of the PVN and at A2.9–2.3 mm for microdissection of the PFLH and ARC, as previously described (Chang et al., 2008). These dissections were stored in RNAlater (Sigma-Aldrich) until processed.

### Quantitative real-time polymerase chain reaction (qRT-PCR)

Dissected brain regions were analyzed for neuropeptide expression. The mRNA from each microdissected sample was purified using a Qiagen RNeasy kit (Qiagen, Valencia, CA), and cDNA was synthesized using high capacity RNA-to-cDNA Master Mix (Life Technologies, Grand Island, NY). A SYBR Green PCR core reagents kit (Life Technologies, Grand Island, NY) was used for qRT-PCR and performed in MicroAmp Optic 96-well Reaction Plates (Life Technologies, Grand Island, NY), under the condition of 2 min at 50°C, 10 min at 95°C, and 40 cycles of 15 s at 95°C and 1 min at 60°C, as previously described (Poon et al., 2012). The levels of target gene expression were quantified relative to the level of cyclophilin-A, using the relative quantification method. Primers were designed with the NCBI Primer design tool (http://www.ncbi.nlm.nih.gov/tools/primer-blast/) to span an exon-exon gap to eliminate amplification of genomic DNA. The primers used were: corticotrophin-releasing factor (CRF) forward: 5′-GCTCAGCAAGCTCACAGCAA-3′, reverse: 5′- GGCCAAGCGCAACATTTC-3′; CXCL12 forward: 5′- AGTGACGGTAAGCCAGTCAGCCT-3′, reverse: 5′-TGACGTTGGCTCTGGCGACA-3′; CXCR4 forward: 5′-GGGCTGGAGAGCGAGCATTGC-3′, reverse: 5′-AAGCAGGGTTCCTTGTTGGAGTCA-3′; CXCR7 forward: 5′-GCCGCGAGGTCACTTGGTTG-3′, reverse: 5′-CAGGGCCAGTTGATGTCCGAGTA-3′. The primers for CRF, ENK, OX, MCH, NPY, and CYC were designed as previously described (Chang et al., 2004). The specificities of PCR products were confirmed by a single band of corresponding molecular weight revealed by agarose gel electrophoresis. The concentration of all target primers was 100 nM, and the CYC primer was 200 nM.

### Immunofluorescence histochemistry

Offspring were sacrificed at P15 via perfusion, as previously described (Chang et al., 2008), and their brains were removed, immediately fixed in 4% paraformaldehyde at 4°C for 48–72 h, and then cryo-protected in 25% sucrose at 4°C for 60–72 h. Serial, 30 μm coronal sections of the hypothalamus were cut with a cryostat, and every third section was collected for immunofluorescence histochemistry analysis of the neuronal marker NeuN and the cellular proliferation marker Ki67. The free-floating sections were incubated first with chicken anti-NeuN (1:500; EMD Millipore, Billerica, MA) and rabbit anti-Ki67 (1:500; Life Technologies, Grand Island, NY) followed by secondary antibodies goat anti-chicken Alexafluor-488 (1:400; Life Technologies) and goat anti-rabbit Alexafluor-594 (1:400; Life Technologies). Fluorescence images were captured using a Zeiss Axioplan 2 microscope (Zeiss, Thornwood, NY) and blind analyzed. The cell densities of NeuN/Ki67 were collected from 4 to 5 sections per brain in all five groups at the same anterior-posterior levels relative to Bregma (PVN, −1.08 to −2.04 mm), and the average cell densities in each group were quantified with ImageJ (http://imagej.nih.gov/ij/). With the densities of cells that double-labeled NeuN with Ki67 yielding similar results whether relative to NeuN or Ki67, only the data relative to NeuN is reported in the manuscript. Triple-labeling of Ki67 and NeuN with ENK was not performed due to the low labeling of this neuropeptide and the use of colchicine to enhance ENK labeling known to induce neuronal changes of its own that would complicate the interpretation of the results (Shaughnessy et al., 1998; Pu et al., 1999; Yan and Ribak, 1999).

### Behavioral testing

Behavioral testing was conducted in a sound-attenuated room under low ambient red light, starting 45 min into the dark cycle. First, rats starting at P60 were tested for novelty-induced locomotor activity (activity on first exposure) in an open field activity chamber for 15 min. Each rat was placed in a novel 43.2 × 43.2 × 30.5 cm activity test chamber (Med Associates, Inc., St. Albans, VT), while measures of ambulatory distance, ambulatory time, ambulatory counts (number of infrared beam breaks), ambulatory episodes, and resting time were recorded. Following this test, the rats were given three additional exposures to the chambers over three consecutive days, and on the following day, they were tested for novelty-seeking with a novel object (a 2 × 3 in block), with the amount of time spent in the quadrant containing the object automatically recorded. The rats were then tested 11 days later for anxiety in an elevated plus maze. They were placed in the center of the maze, alternately facing an open or closed arm, and left in the maze for 5 min while videotaped. The maze consisted of four arms (10 × 50 cm each), 55 cm above the floor, with two opposite arms enclosed by 30 cm-high opaque walls. An observer blind to treatment condition later scored the videotape for time in arms and number of arm entries, with the criterion for entry being both forepaws into an arm.

### Data analysis

Differences between the effects of all prenatal treatments on locomotor behavior were assessed using a two-way repeated-measures ANOVA with diet/drug/dose as a repeated measure, followed by a one-way ANOVA for each treatment and Tukey's *post-hoc* test. Elevated plus maze comparisons were made using a one-way ANOVA, followed by Tukey's *post-hoc* test. To determine significant differences between the 5 independent groups on caloric intake and body weight over time, a Kruskal-Wallis test was used, followed by paired, two-tailed *t*-tests when appropriate. For experiments examining gene expression, a one-way ANOVA was used for each neuropeptide or chemokine, followed up by a Tukey's *post-hoc* test when appropriate. Significance was determined at *p* < 0.05. Data are reported as mean ± standard error of the mean (S.E.M.). Since in all experiments *post hoc* comparisons of saline injection to the no treatment control failed to reveal any effect of the injection itself on any of the tests administered (see Results section), all comparisons of the HFD and CXCL12 treatment groups were made against the no treatment control.

## Results

### Consumption of a HFD increases circulating levels of CXCL12 in pregnant rats

**Figure 1:**
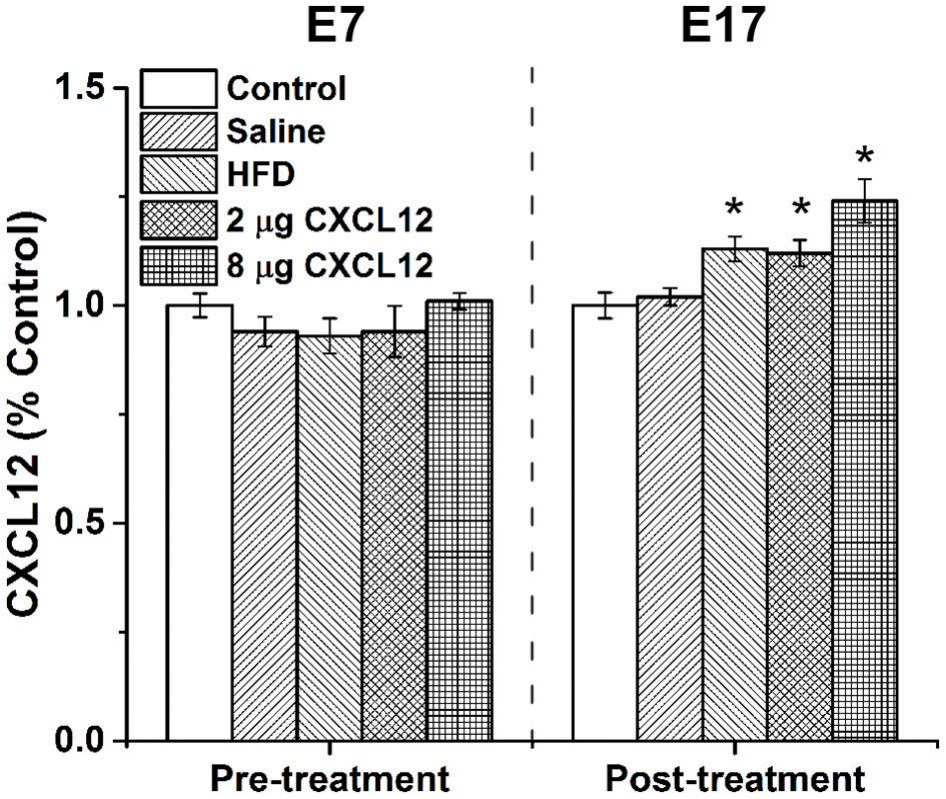
**HFD or CXCL12 exposure increases circulating CXCL12 levels in pregnant rats**. Daily treatment of either a HFD or CXCL12 at 2 or 8 μg in dams beginning at embryonic day 7 (E7) led to a significant increase in circulating CXCL12 levels by E17 as compared to control. ^*^p < 0.05 vs. control.

Consumption of a HFD by adult humans and animals has been shown to increase circulating inflammatory mediators in blood (Milanski et al., 2009; Gregor and Hotamisligil, 2011). This experiment tested whether HFD consumption by pregnant rats increases circulating levels of the chemokine, CXCL12, in the dam and then determined the dose of peripherally injected CXCL12 that produces an equivalent increase in circulating CXCL12. Tail vein blood collected from dams at E7, prior to any treatment, showed that the different treatment and control groups used in this experiment were similar in their baseline levels of plasma CXCL12 [*F*_(4, 33)_ = 1.91, *p* = 0.13] (Table 1). Blood collected from these dams at E17 after treatment, however, revealed a significant main effect on CXCL12 levels across groups [*F*_(4, 33)_ = 8.91, *p* < 0.01]. Compared to the control group, there was a significant increase in circulating CXCL12 levels in dams consuming the HFD (13%; *p* < 0.05) or injected with CXCL12 at 2 μg (12%; *p* < 0.05) or at 8 μg (24%; *p* < 0.01), with no difference detected between the saline injection and control dams (see Data Analysis in Methods, Figure 1, Table 1). While the HFD and 2 μg CXCL12 groups showed no difference in their circulating CXCL12 levels (*p* = 0.84), the change in the 8 μg CXCL12 group was 2-fold higher (*p* < 0.05). These results demonstrate that HFD consumption increases circulating levels of CXCL12 in pregnant dams and that injection of CXCL12 at 2 μg elevates circulating CXCL12 to the same level as the HFD while the 8 μg dose has a significantly greater effect.

**Table 1:** **Average circulating levels of CXCL12 in dams pre- and post-treatment**.

	**Pre-treatment (ng/mL)**	**Post-treatment (ng/mL)**
Control	1.98 ± 0.05	1.85 ± 0.06
Saline injection	1.85 ± 0.06	1.94 ± 0.04
HFD	1.84 ± 0.07	2.08 ± 0.06^*^
2 μg CXCL12	1.87 ± 0.11	2.06 ± 0.07^*^
8 μg CXCL12	2.01 ± 0.03	2.29 ± 0.11^*^

* *p < 0.05, as compared to negative control*.

### Prenatal HFD exposure affects expression of CXCL12 chemokine system in the hypothalamus of offspring

**Figure 2:**
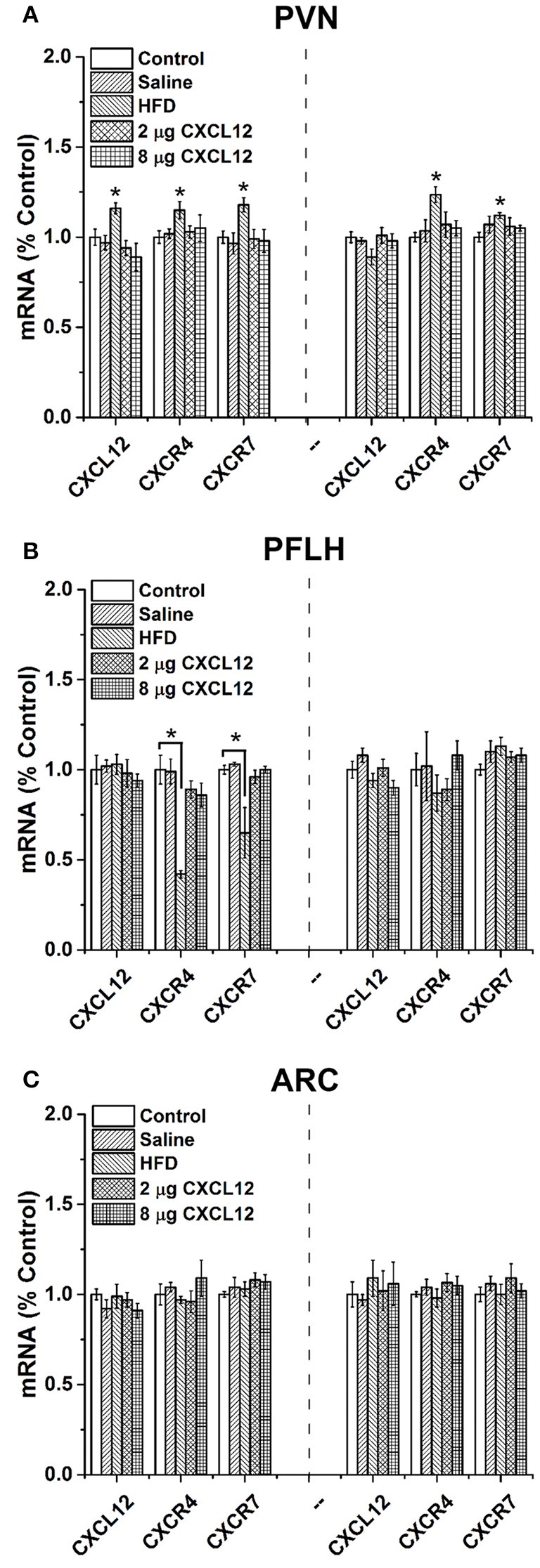
**Prenatal HFD affects CXCL12 chemokine system in the hypothalamus of offspring**. The graphs represent the expression of CXCL12, CXCR4, and CXCR7 at postnatal day 15 (P15) (left) and P30 (right). **(A)** In the paraventricular nucleus (PVN), a significant increase of CXCL12, CXCR4, and CXCR7 was found with prenatal HFD exposure at both P15 and P30, with no effect seen with 2 or 8 μg CXCL12 or saline as compared to control. **(B)** In the perifornical lateral hypothalamus (PFLH), a significant decrease in CXCR4 and CXCR7 was found with prenatal HFD at P15, with no effect at P30 or on CXCL12, and there was no effect with prenatal exposure to 2 or 8 μg CXCL12 as compared to control. **(C)** In the arcuate nucleus (ARC), no effect on the expression of CXCL12, CXCR4, or CXCR7 was found in any of the treatment groups compared to control at either P15 or P30. ^*^p < 0.05 vs. control.

In addition to increasing circulating CXCL12, our recent study in adult rats demonstrated that HFD consumption significantly increases the expression of CXCL12 and its receptors in the hypothalamus, with this effect occurring specifically in the PVN and PFLH but not in the ARC (Poon et al., 2016). In the present experiment, we tested whether HFD intake in pregnant rats has similar effects on the hypothalamic CXCL12 system of the offspring and whether this central system is affected by peripheral injection of CXCL12 itself. The offspring from 5 groups of dams were examined at two postnatal ages, P15 and P30, for expression of CXCL12, CXCR4, and CXCR7 in the PVN, PFLH and ARC, and they exhibited changes with prenatal HFD exposure compared to control (Figure 2A) that were generally similar to those seen in adult rats consuming a HFD (Poon et al., 2016). In the PVN, a significant main effect of treatment on the expression of CXCL12 in the offspring was found at P15 [*F*_(4, 28)_ = 3.55, *p* < 0.05] but not P30 [*F*_(4, 28)_ = 1.75, *p* = 0.17], with *post-hoc* tests showing prenatal HFD exposure to significantly increase CXCL12 expression only at P15 (16%; *p* < 0.05). Also in the PVN, a significant main effect of treatment was found at P15 on the expression of CXCR4 [*F*_(4, 28)_ = 2.14, *p* < 0.05] and CXCR7 [*F*_(4, 28)_ = 2.96, *p* < 0.05], with *post hoc* tests showing prenatal HFD exposure to significantly increase the expression of both CXCR4 (15%; *p* < 0.01) and CXCR7 (18%; *p* < 0.01). These HFD-induced changes in the receptors persisted at P30, again with a significant main effect of treatment on the expression of CXCR4 [*F*_(4, 28)_ = 3.22, *p* < 0.05] and CXCR7 [*F*_(4, 28)_ = 1.91, *p* < 0.05] reflecting a significant increase in both CXCR4 (24%; *p* < 0.01) and CXCR7 (12%; *p* < 0.01) mRNA. Very different results were obtained in the two other hypothalamic areas examined. In the PFLH (Figure 2B), prenatal HFD exposure had no effect on the expression of CXCL12 at P15 [*F*_(4, 28)_ = 0.25, *p* = 0.91] or P30 [*F*_(4, 28)_ = 2.51, *p* = 0.06], and a significant main effect of treatment on CXCR4 [*F*_(4, 28)_ = 3.03, *p* < 0.05] and CXCR7 [*F*_(4, 28)_ = 4.55, *p* < 0.01] was shown by *post hoc* tests to reflect a HFD-induced decrease in expression of CXCR4 (−59%; *p* < 0.01) and CXCR7 (−35%; *p* < 0.01) at P15, with no effect evident at P30 for CXCR4 [*F*_(4, 28)_ = 0.88, *p* = 0.49] or CXCR7 [*F*_(4, 28)_ = 1.44, *p* = 0.25]. Further, in the ARC (Figure 2C), prenatal HFD exposure had no effect on CXCL12 at P15 [*F*_(4, 27)_ = 0.83, *p* = 0.52] or P30 [*F*_(4, 28)_ = 0.46, *p* = 0.77], on CXCR4 at P15 [*F*_(4, 26)_ = 1.70, *p* = 0.18] or P30 [*F*_(4, 28)_ = 0.65, *p* = 0.63], or on CXCR7 at P15 [*F*_(4, 26)_ = 0.62, *p* = 0.65] or P30 [*F*_(4, 28)_ = 0.69, *p* = 0.61]. In contrast to these significant effects of prenatal HFD exposure, peripheral injection of CXCL12 into the pregnant rats had no effect on the endogenous CXCL12 system. It produced no change in the expression of CXCL12, CXCR4, or CXCR7 in the PVN, PFLH or ARC, at either dose tested (2 or 8 μg) or either age tested (P15 or P30; *p* > 0.05) (Figures 2A–C). Together, these results show that prenatal exposure to a HFD, in addition to elevating circulating CXCL12 as shown in the first experiment, has a significant stimulatory effect on CXCL12 and its receptors in the PVN, but not the PFLH or ARC, while prenatal exposure to CXCL12 elevated to the same levels induced by a HFD has no effect on the development of its own endogenous system.

### Prenatal HFD and CXCL12 exposure affect neuropeptides in the hypothalamus of offspring

**Figure 3:**
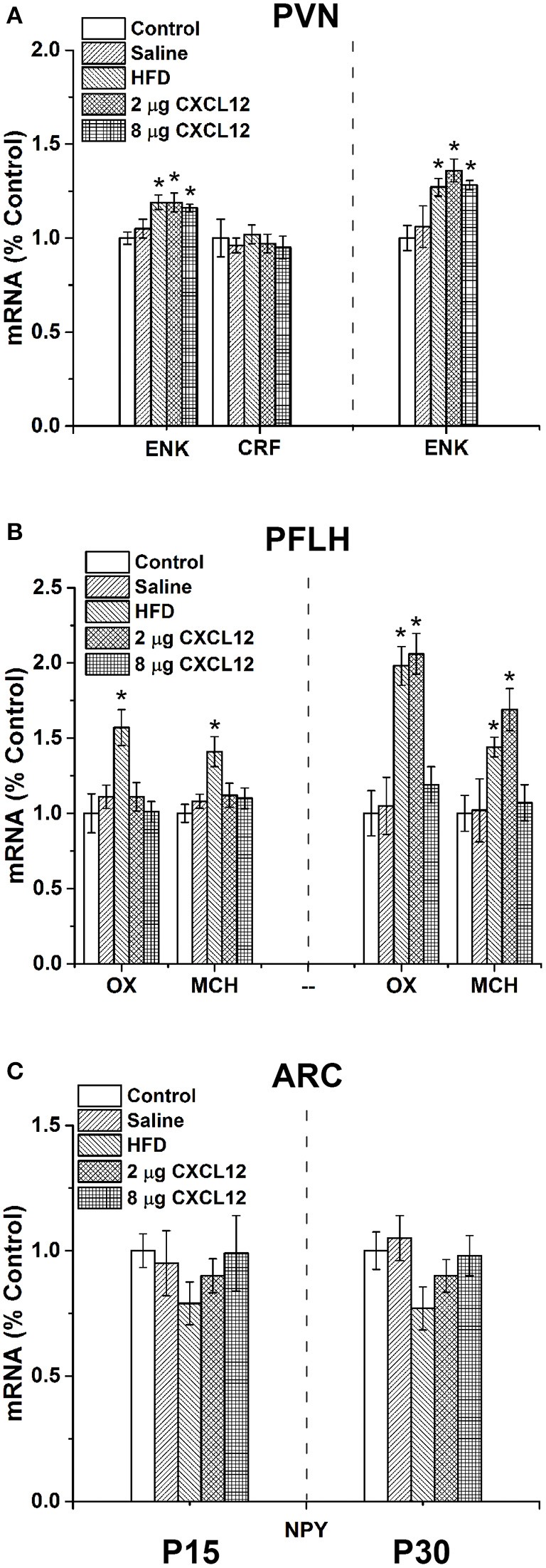
**Prenatal HFD and CXCL12 affect neuropeptides in the hypothalamus of offspring**. The graphs represent the expression of neuropeptides at postnatal day 15 (P15) (left) and P30 (right). **(A)** In the PVN, a significant increase of enkephalin (ENK) was found with both prenatal HFD exposure and CXCL12 at 2 and 8 μg at P15 and P30, with no effect of saline as compared to control. No effect was seen on corticotrophin-releasing factor (CRF) at P15. **(B)** In the PFLH, a significant increase in orexin (OX) and melanin-concentrating hormone (MCH) was found with prenatal HFD exposure at P15 and P30, but there was no effect of prenatal exposure to CXCL12 at 8 μg in any age group or at 2 μg as compared to control, with only the 2 μg dose at P30 significantly increasing the expression of OX and MCH. **(C)** In the ARC, no effect was found on the expression of neuropeptide Y (NPY) in any of the groups at either P15 or P30. ^*^p < 0.05 vs. control.

Prior studies of the hypothalamic peptides have shown that prenatal HFD exposure stimulates ENK expression in the PVN and OX and MCH in the PFLH of postnatal offspring at both P15 and P30, while having no effect on or suppressing NPY in the ARC (Chang et al., 2008). They also show that CXCL12 injection in adult rats similarly stimulates the expression of ENK in the PVN but has no effect on OX or MCH in the PFLH (Poon et al., 2016). This experiment tests and compares the effects of prenatal HFD exposure and prenatal CXCL12 exposure on neuropeptide expression in the offspring's hypothalamus. In the PVN, the results reveal similar effects of these two treatments on ENK (Figure 3A). At P15, a significant main effect was found of treatment on the expression of ENK [*F*_(4, 27)_ = 3.48, *p* < 0.05], with *post hoc* tests showing a significant increase in ENK with prenatal exposure to HFD (19%; *p* < 0.01), 2 μg CXCL12 (19%; *p* < 0.05), and 8 μg CXCL12 (16%; *p* < 0.01) as compared to control. Further measurements of the stress-related peptide, CRF, revealed no difference between the groups [*F*_(4, 7)_ = 0.20, *p* = 0.94]. Similarly at P30, a significant main effect of treatment on ENK expression in the PVN was evident [*F*_(4, 28)_ = 3.68, *p* < 0.01], with *post hoc* tests again revealing a significant increase in ENK with prenatal exposure to HFD (27%; *p* < 0.01), 2 μg CXCL12 (37%; *p* < 0.01), and 8 μg CXCL12 (28%; *p* < 0.01). In the PFLH and ARC, very different results were once again obtained. In the PFLH (Figure 3B), a significant main effect was found for treatment on expression of OX in the PFLH at both P15 [*F*_(4, 27)_ = 3.38, *p* < 0.05] and P30 [*F*_(4, 28)_ = 3.87, *p* < 0.01]. *Post hoc* tests revealed with prenatal HFD exposure a significant increase in OX expression at both P15 (57%; *p* < 0.05) and P30 (98%; *p* < 0.01), as shown previously (Chang et al., 2008), but no effect of CXCL12 injection on OX expression at 2 or 8 μg in P15 offspring or at 8 μg in P30 offspring, with only the 2 μg dose at P30 significantly increasing OX (106%; *p* < 0.01). Similarly, a significant main effect was also found for treatment on MCH expression in the PFLH at both P15 [*F*_(4, 28)_ = 2.83, *p* < 0.05] and P30 [*F*_(4, 28)_ = 3.70, *p* < 0.05]. Also, *post hoc* tests revealed with prenatal HFD exposure a significant increase in expression of MCH at both P15 (41%; *p* < 0.05) and P30 (44%; *p* < 0.01), but no change in MCH after injection of CXCL12 at 2 and 8 μg at P15 or at 8 μg at P30, with only the 2 μg dose at P30 increasing MCH expression in offspring (69%; *p* < 0.01). In the ARC, the expression of NPY was unaffected by any treatment (Figure 3C). Although there was a tendency for prenatal HFD exposure to decrease the expression of NPY as suggested by prior studies (Chang et al., 2008), a statistical analysis failed to reveal a significant change in this peptide with any treatment compared to control, at both P15 [*F*_(4, 28)_ = 1.17, *p* = 0.35] and P30 [*F*_(4, 28)_ = 2.37, *p* = 0.08]. While confirming prior studies showing that prenatal HFD exposure stimulates the expression of ENK in the PVN and of OX and MCH in the PFLH while having little effect on NPY in the ARC, the results here demonstrate that prenatal CXCL12 exposure mimics the stimulatory effect of prenatal HFD on ENK in the PVN at both doses and both ages of postnatal offspring but increases the expression of OX and MCH in the PFLH only at the 2 μg dose and at the P30 age while having no effect on expression of NPY in the ARC at any dose or age. These findings distinguish the PVN as a site where this chemokine may be most active.

### Prenatal HFD and CXCL12 increase neurogenesis in the PVN of offspring

**Figure 4:**
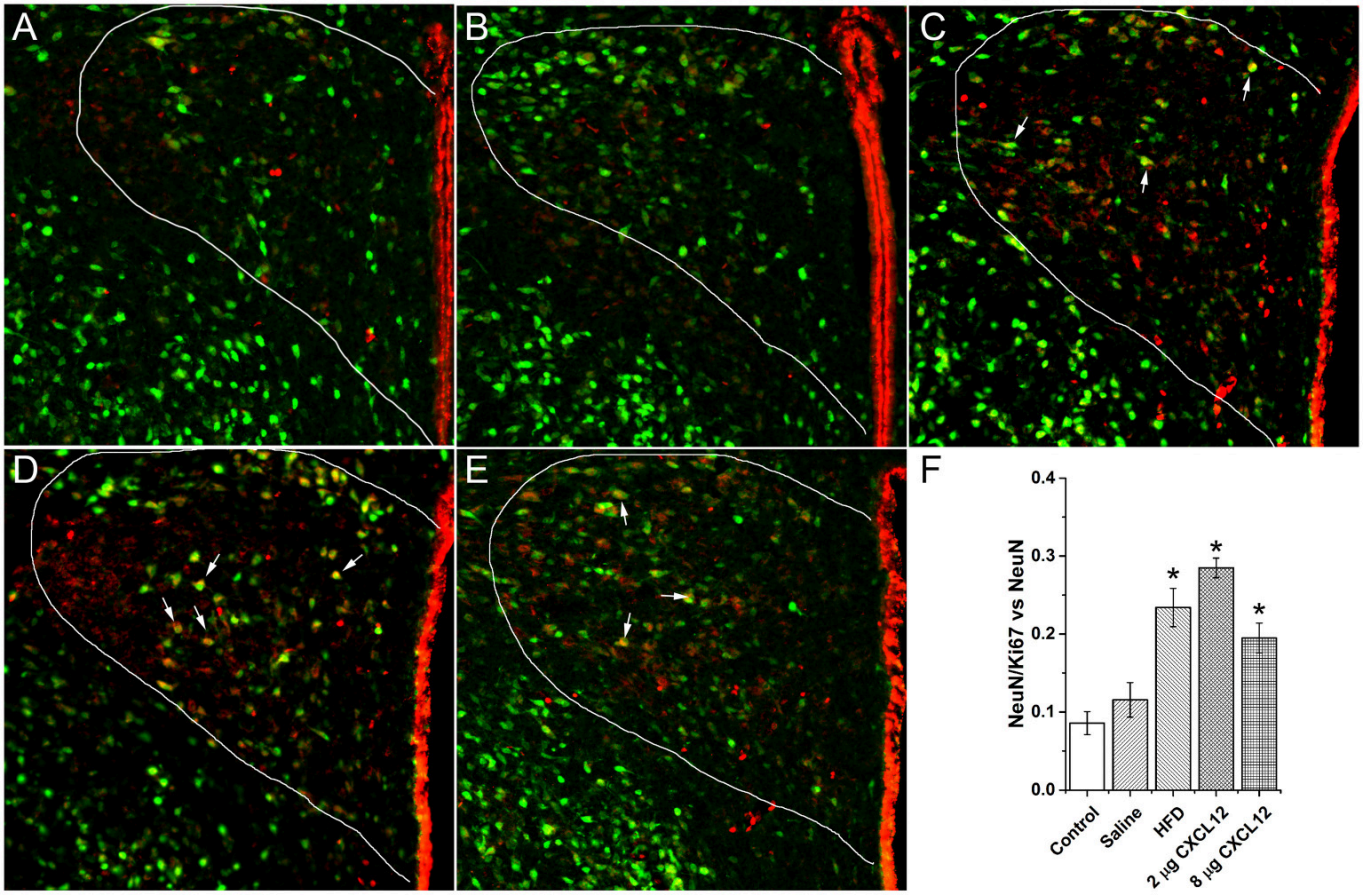
**Prenatal HFD and CXCL12 increased neurogenesis in the PVN**. Representative photomicrographs of neurons double-labeled with NeuN and Ki67 from the PVN of P15 offspring under the following prenatal conditions: **(A)** negative control rats; **(B)** saline injected rats; **(C)** HFD exposed rats; **(D)** 2 μg CXCL12 exposed rats; and **(E)** 8 μg CXCL12 exposed rats. The white arrows depict neurons positive for both NeuN and Ki67. **(F)** Prenatal exposure to a HFD, 2 or 8 μg CXCL12, significantly increased the number of double-labeled NeuN/Ki67 neurons as compared to control. ^*^p < 0.05 vs. control.

In addition to stimulating neuropeptides, prenatal exposure to a HFD has previously been shown to increase neurogenesis in the hypothalamus of early postnatal offspring (Chang et al., 2008). With our results identifying the PVN as an important site where prenatal HFD and CXCL12 have similar actions on neuropeptide development, this experiment examined this site and tested whether prenatal HFD and CXCL12 exposure are also similar in increasing neuronal proliferation in the PVN. Using immunofluorescence double-labeling with Ki67 as a proliferative marker and NeuN as a neuronal marker, analyses of the PVN in P15 offspring revealed a main effect of treatment on the number of double-labeled Ki67/NeuN cells [*F*_(4, 20)_ = 18.98, *p* < 0.001], with *post hoc* tests showing a significantly greater number of new neurons as compared to control for the HFD (*p* < 0.001), 2 μg CXCL12 (*p* < 0.001), and 8 μg CXCL12 (*p* < 0.01) (Figure 4). These results demonstrate that prenatal CXCL12 and HFD are similar in stimulating neurogenesis in the PVN, consistent with the finding that they both increase ENK expression in this brain region.

### Prenatal HFD and CXCL12 exposure similarly alter emotional behaviors in offspring

**Figure 5:**
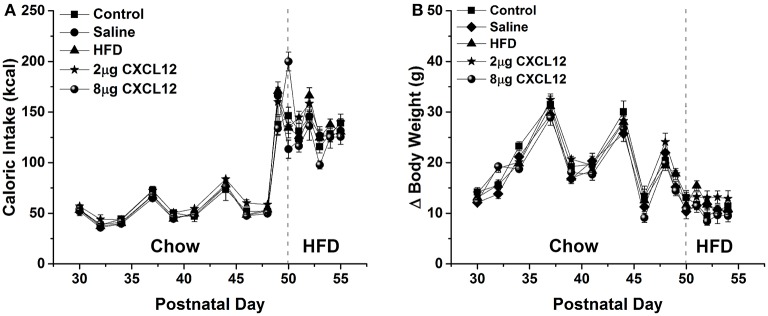
**Prenatal HFD and CXCL12 had no effect on caloric intake and body weight**. The left of the dashed line represents maintenance on chow from P30-48 as measured every 2–3 days, while the right of the dashed line represents daily measurements of a 7-day HFD challenge from P50 to P57. **(A)** No effect in any of the treatment groups compared to control was seen in caloric intake during both the maintenance period and on the 7-day HFD challenge. **(B)** No effect in any of the treatment groups compared to control was seen on change in body weight during both the maintenance period and the 7-day HFD challenge.

**Figure 6:**
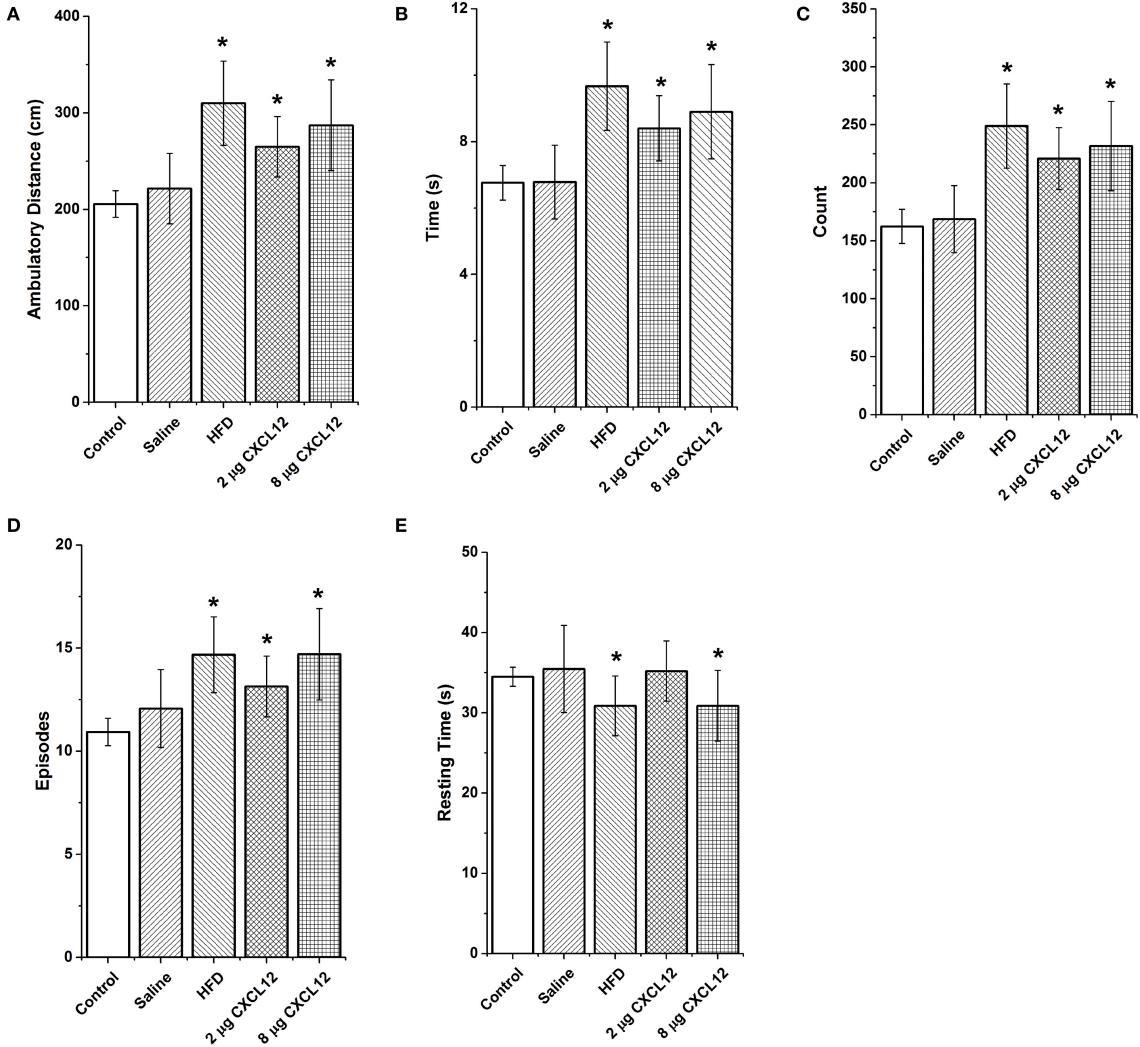
**Prenatal HFD and CXCL12 similarly increase locomotor activity in a novel environment**. Prenatal exposure to a HFD or CXCL12 at 2 or 8 μg compared to control significantly increases locomotor activity as measured by **(A)** ambulatory distance; **(B)** ambulatory time; **(C)** ambulatory counts (number of infrared beam breaks); and **(D)** ambulatory episodes. **(E)** A decrease in resting time was found in the prenatal HFD and 8 μg CXCL12 offspring but not the 2 μg CXCL12 as compared to control offspring. ^*^p < 0.05 vs. control.

**Figure 7:**
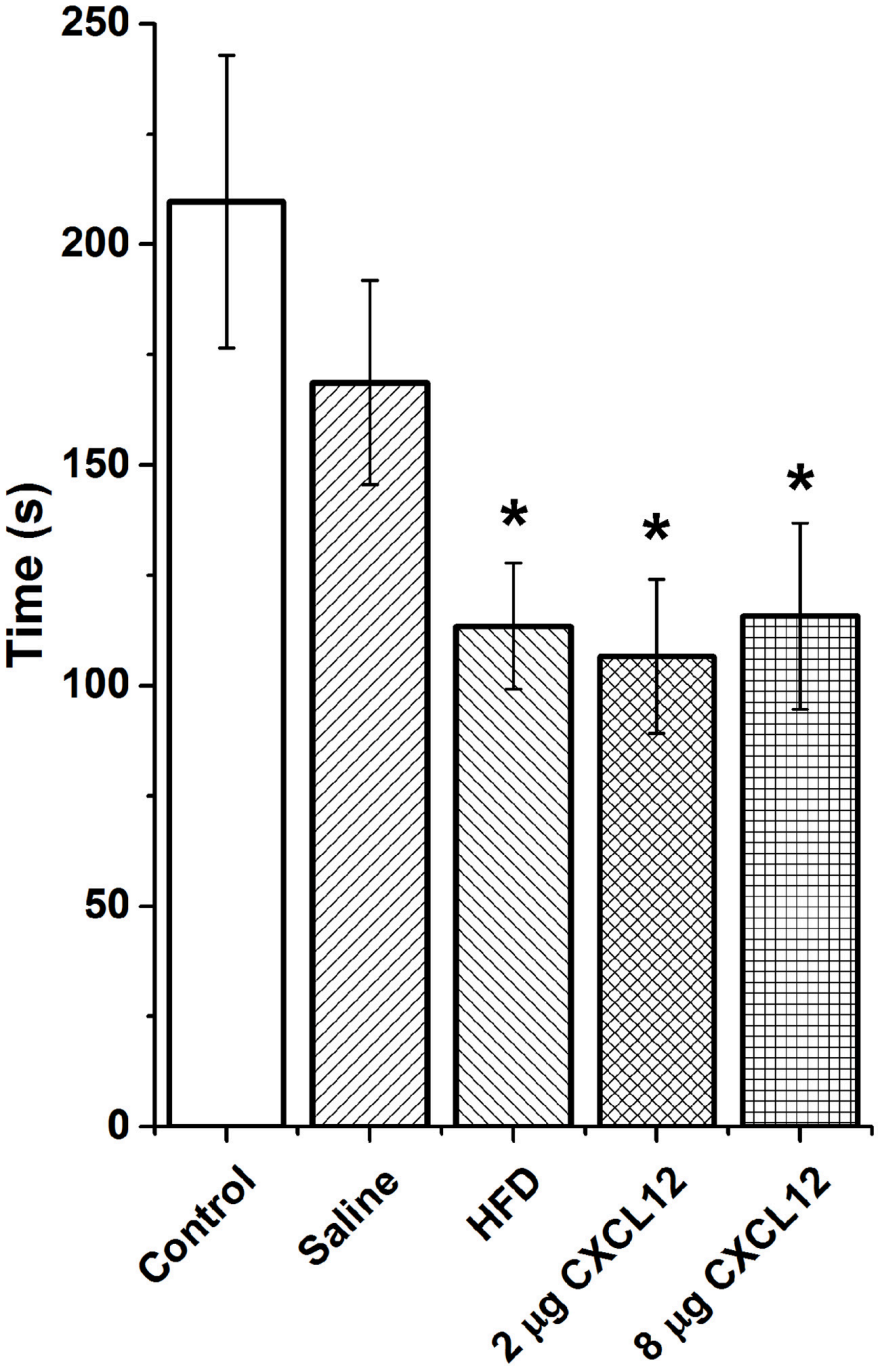
**Prenatal HFD and CXCL12 similarly reduce novelty-seeking**. Prenatal exposure to a HFD or CXCL12 at 2 or 8 μg caused a significant decrease in the time spent in the quadrant with a novel object as compared to control offspring. ^*^p < 0.05 vs. control.

**Figure 8:**
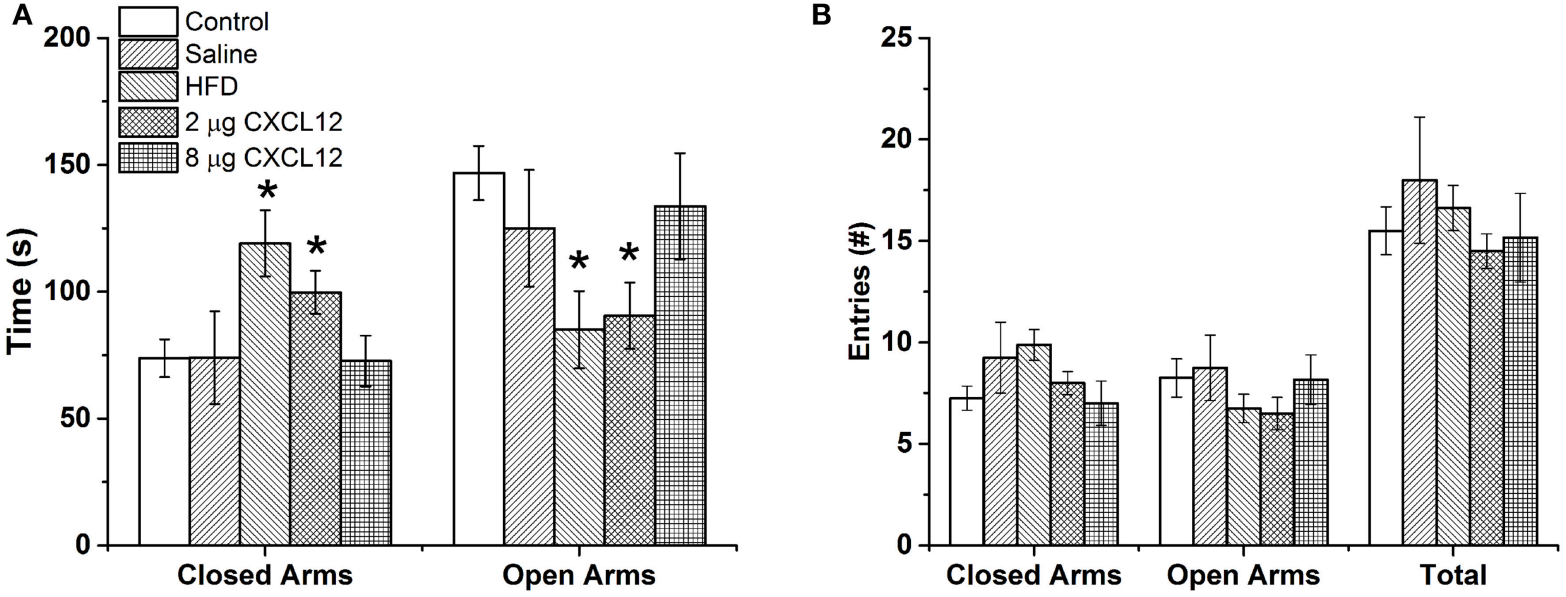
**Prenatal HFD and CXCL12 similarly increase anxiety-like behavior. (A)** In an elevated plus maze, prenatal exposure to a HFD or CXCL12 at 2 μg, but not 8 μg, caused a significant decrease in time spent in the open arms and increase in time spent in the closed arms as compared to control offspring. **(B)** No differences in locomotor activity were found as measured by the number of entries into the open arms, closed arms, or total. ^*^p < 0.05 vs. control.

Changes in ENK in the PVN have been linked to an increase in anxiety as well as other emotional behaviors (Chang et al., 2010; Barson et al., 2013; Melo et al., 2014). The question addressed in this experiment is whether the similar effects of prenatal HFD and prenatal CXCL12 exposure on hypothalamic neurogenesis and neuropeptide gene expression are accompanied by significant changes in the offspring's behavior. Although all offspring showed an increase in HFD intake over chow when first introduced, there was no effect of prenatal HFD or prenatal CXCL12 treatment on the measures of caloric intake, HFD preference, or body weight in the offspring (Figure 5). While a Kruskal-Wallis test showed at a few postnatal ages a significant difference in caloric intake and body weight change during maintenance on chow from P30–P48 (Table 2), *post hoc* tests did not reveal any significant differences between the groups as compared to control at any of the time points (*p* > 0.05). Further, while the 7-day HFD challenge with chow present from P50–57 also revealed a significant difference in caloric intake and body weight change at a few time points (Table 3), *post hoc* tests showed no significant group differences in caloric intake or preference for the HFD at any of the time points (*p* > 0.05). In contrast to consummatory behavior, measures of the emotional behaviors in the offspring at P50 revealed significant changes that were similar after the prenatal HFD and prenatal CXCL12 treatments. In the novel activity chamber, prenatal treatment significantly affected measures of activity [*F*_(1, 4)_ = 1625.48, *p* < 0.01], with a significant interaction effect detected between group and activity [*F*_(4, 64)_ = 1002.52, *p* < 0.01]. Further analyses revealed a main effect of treatment on ambulatory distance [*F*_(4, 35)_ = 3.67, *p* < 0.05], ambulatory time [*F*_(4, 35)_ = 3.66, *p* < 0.05], ambulatory counts [*F*_(4, 35)_ = 3.45, *p* < 0.05], ambulatory episodes [*F*_(4, 35)_ = 4.72, *p* < 0.01] and resting time [*F*_(4, 35)_ = 4.62, *p* < 0.01], reflecting a significant increase after prenatal exposure to a HFD or to CXCL12 at the 2 and 8 μg doses compared to control in the measures of novelty-induced locomotor activity, ambulatory distance, ambulatory time, ambulatory counts, and ambulatory episodes (*p* < 0.05) and significant decrease in resting time after prenatal exposure to a HFD or to CXCL12 at 8 μg (*p* < 0.05) (Figure 6). In the familiar activity chamber, the addition of a novel object had a significantly different effect on novelty-seeking behavior between the different prenatal treatment groups [*F*_(4, 33)_ = 4.21, *p* < 0.01], with *post hoc* tests revealing a decrease in the amount of time spent in the quadrant with the novel object for offspring prenatally exposed to a HFD (*p* < 0.01) or to CXCL12 at 2 μg (*p* < 0.01) and 8 μg (*p* < 0.01) (Figure 7). Further, in the elevated plus maze, the different groups showed a significant difference in time spent in both the open arms [*F*_(4, 34)_ = 5.53, *p* < 0.01] and closed arms [*F*_(4, 34)_ = 3.74, *p* < 0.01]. The prenatal HFD offspring exhibited a significant decrease in time spent in the open arms (*p* < 0.01) and an increase in time spent in the closed arms (*p* < 0.01), and similarly, prenatal CXCL12 at 2 μg significantly decreased the time spent in the open arms (*p* < 0.01), and increased the time spent in the closed arms (*p* < 0.05) (Figure 8A), while the 8 μg CXCL12 dose produced no significant change in any of these measures (*p* > 0.05). There was no effect on spontaneous motor behavior in the maze as measured by closed arm entries [*F*_(4, 34)_ = 1.14, *p* = 0.36], open arm entries [*F*_(4, 34)_ = 1.10, *p* = 0.38], and total arm entries [*F*_(4, 34)_ = 1.21, *p* = 0.32] (Figure 8B). These results demonstrate that prenatal exposure to a HFD or to CXCL12 has similar anxiety-like effects on the postnatal offspring.

**Table 2:** **Kruskal-wallis test of significance of caloric intake and change in body weight during the chow maintenance period**.

	**Caloric Intake (Chow)**		Δ **Body Weight (g)**	
	**H(4)**	***p***	**H(4)**	***p***
P30	6.92	0.14	11.54	<0.05^*^
P32	7.18	0.13	21.02	<0.01^^**^^
P34	8.62	0.07	13.09	<0.05^*^
P37	7.95	0.09	4.49	0.34
P39	9.55	<0.05^*^	10.46	<0.05^*^
P41	10.71	<0.05^*^	2.51	0.64
P44	5.23	0.26	3.34	0.50
P46	13.01	<0.05^*^	7.75	0.10
P48	11.87	<0.05^*^	6.98	0.14

* *p < 0.05*,

** *p < 0.01*.

**Table 3:** **Kruskal-wallis test of significance of caloric intake and change in body weight during the 7-day HFD challenge**.

	**Caloric Intake (HFD** + **Chow)**		Δ **Body Weight (g)**	
	**H(4)**	***p***	**H(4)**	***p***
P49	9.56	<0.05^*^	–	–
P50	19.27	<0.01^^**^^	9.72	<0.05^*^
P51	9.09	0.06	4.22	0.38
P52	10.72	<0.05^*^	9.33	<0.05^*^
P53	12.01	<0.05^*^	7.2	0.13
P54	2.65	0.62	3.91	0.42
P55	2.79	0.89	3.97	0.41

* *p < 0.05*,

** *p < 0.01*.

## Discussion

Evidence reveals a strong link between HFD intake and inflammation (Milanski et al., 2009; Gregor and Hotamisligil, 2011; Reynolds et al., 2014), with our recent report in adult rats focusing attention on the CXCL12 chemokine system and its relation to dietary fat and the neuropeptide ENK in the PVN (Poon et al., 2016). The present study investigated a possible association between the CXCL12 system and the stimulatory effects of HFD exposure during gestation on hypothalamic neuropeptides and behavior in the offspring. The results demonstrate several new findings: (1) maternal consumption of a HFD, in addition to increasing circulating CXCL12 in the dam, also stimulates the expression of CXCL12 and its receptors, CXCR4 and CXCR7, in the PVN of the postnatal offspring; (2) peripheral injection of CXCL12 in the dams elevates circulating CXCL12 to similar levels as a HFD; (3) peripheral CXCL12 injection acts similarly to prenatal HFD exposure in stimulating the expression of ENK in the PVN, increasing neurogenesis in this nucleus, and increasing anxiety-related behaviors in postnatal offspring; and (4) CXCL12 in the dam has a weaker or no effect on the expression of other neuropeptides, namely, OX, and MCH in the PFLH which are stimulated by a HFD but only by one dose of CXCL12 at one age and NPY in the ARC, which is unaffected by both the HFD and CXCL12. These results suggest that the CXCL12 chemokine system is most active in the PVN where it may function in close relation to a HFD in stimulating the development of peptide neurons and producing changes in emotional behavior in postnatal offspring.

### Consumption of a HFD increases circulating levels of CXCL12 in pregnant rats

The consumption of a HFD is known to induce systemic inflammation, causing an increase in levels of several inflammatory markers such as the cytokines, TNF-α and IL-6 (Takanabe-Mori et al., 2010). In a recent study (Poon et al., 2016), a 5-day HFD challenge on CXCL12 in adult rats induced a similar increase in circulating levels of this chemokine. There are few studies examining the effects of HFD ingestion during pregnancy on inflammation. The results of the present study demonstrate in pregnant rats that consumption of a HFD during the critical period of hypothalamic neuronal development (from E9 to E18) significantly increases circulating levels of CXCL12 in the dam and that peripheral injections of CXCL12 over the same time course causes a similar increase at a dose of 2 μg and 2-fold greater increase at an 8 μg dose. In addition to confirming in pregnant rats the stimulatory effect of HFD consumption on systemic inflammation, these results reveal a dose of daily peripherally injected CXCL12 that increases circulating CXCL12 to the same level as the HFD, thus providing a means for testing whether an inflammatory mediator such as this chemokine can mimic the effects of the HFD condition on the pregnant rat and offspring.

### Prenatal HFD exposure stimulates expression of CXCL12 chemokine system in the PVN of offspring

Studies of the brain have revealed a broad, overall effect of prenatal HFD exposure on inflammation, including an increase in inflammatory markers in the hypothalamus (Grayson et al., 2010) as well as in adipose tissue (Murabayashi et al., 2013) and other organs (Wallace et al., 2001). In addition to increasing circulating levels of CXCL12 in dams, the present study showed the HFD to affect CXCL12 and its receptors, CXCR4 and CXCR7, in the offspring's hypothalamus, in a site-specific manner. Specifically, it increased the expression of this chemokine and its receptors in the PVN at both ages (P15 and P30), while decreasing CXCR4 and CXCR7 expression in the PFLH only at P15 and having no effect in the ARC at either age. Interestingly, these site-specific effects in the offspring prenatally exposed to a HFD are similar to our prior study in adult male rats, showing HFD intake to stimulate CXCL12, CXCR4, and CXCR7 expression in the PVN while having no effect in the ARC (Poon et al., 2016). They differ in the PFLH, however, where the chemokine system is stimulated by a HFD in adult rats but unaffected in prenatally exposed rats, possibly reflecting the differential sensitivity of adult and fetal brains to different neurotoxins and greater susceptibility of the fetal brain during development (Rice and Barone, 2000; Zoeller et al., 2002). These results focus attention on the PVN, where the local CXCL12 chemokine system is most strongly stimulated by prenatal HFD exposure and possibly functionally related to the stimulatory effect of a HFD on the development of hypothalamic peptide-expressing neurons.

### Prenatal HFD and CXCL12 similarly stimulate neuropeptide expression in the PVN of offspring

Whereas, a close relationship between HFD intake and inflammation (Takanabe-Mori et al., 2010; Poon et al., 2016) and the prenatal HFD effects on hypothalamic neuropeptide expression (Chang et al., 2008) have been thoroughly established, the responsiveness of the offspring's brain to an inflammatory mediator has yet to be described. Our results confirm the stimulatory effect of prenatal HFD exposure on ENK in the PVN and also on OX and MCH in the PFLH, and they are consistent with evidence that NPY in the ARC is unaffected or reduced by a HFD. Using a parallel paradigm to test the effects of CXCL12 itself, we demonstrate here that peripheral injection of CXCL12 during the same period as HFD exposure in pregnancy similarly stimulates ENK in the PVN at both doses and both ages, while stimulating OX and MCH in the PFLH only at one dose and one postnatal age and having no effect on NPY in the ARC. This evidence focuses greater attention on this chemokine system in the PVN, where it may be related to the stimulatory effect of prenatal HFD exposure on ENK. Once again, these results are similar to our previous study, showing that intracerebroventricular injection of CXCL12 in adult rats stimulates ENK in the PVN, while having no effect on the other neuropeptides in other hypothalamic areas (Poon et al., 2016). The possibility that this effect of CXCL12 is mediated by the receptor, CXCR4, is supported by the evidence that this receptor is concentrated in the same hypothalamic region as ENK (Poon et al., 2016) and is consistent with other studies showing activation of this receptor to produce functional changes in neuropeptides and neurotransmitters (Guyon et al., 2005, 2008; Callewaere et al., 2006). The finding that both OX and MCH expression in the PFLH are unaffected by prenatal treatment with CXCL12, except at one dose (2 μg) and one age (P30), suggests a minor role for this chemokine in the PFLH, similar to prior studies showing the firing of MCH neurons to differ in response to CXCL12 treatment at different doses (Guyon et al., 2005). These findings are consistent with the possibility that prenatal exposure to a HFD functions through the CXCL12 chemokine system in the PVN to produce its stimulatory effect on ENK in the offspring.

### Prenatal HFD and CXCL12 increase neurogenesis in PVN of offspring

With prior studies from this lab revealing prenatal exposure to a HFD to increase the proliferation of hypothalamic peptide neurons in both embryos and early prenatal offspring (Chang et al., 2008), this study tested whether prenatal exposure to CXCL12 is similar in inducing this effect. The findings show prenatal CXCL12 to increase the number of proliferative neurons in the PVN of offspring at P15 similar to that induced by prenatal HFD exposure, with these neurons likely to have a variety of neuropeptide phenotypes including ENK. In light of multiple studies demonstrating a role for CXCL12 in controlling neuronal development (Nagasawa et al., 1996; Ma et al., 1998; Mithal et al., 2012), the increase in neurons induced by prenatal exposure to this chemokine suggests that it may be involved in the neurogenesis effects in the hypothalamus induced by prenatal exposure to a HFD.

### Prenatal HFD or CXCL12 similarly alter emotional behaviors in offspring

With prenatal HFD exposure known to increase anxiety-like behaviors in offspring (Bilbo and Tsang, 2010; Sullivan et al., 2010), and ENK in the PVN specifically shown to be a positive factor in inducing anxiety (Bilkei-Gorzo et al., 2008; Csabafi et al., 2011; Melo et al., 2014), this study tested whether prenatal CXCL12 administration has similar behavioral effects as the HFD. The results demonstrate that offspring exposed to a HFD or CXCL12 during the peak period of hypothalamic development exhibit very similar behavioral changes, namely, an increase in locomotor activity in a novel open field, a decrease in novelty-seeking, and a decrease in time spent in the open arms of an elevated plus maze. These behaviors suggest that CXCL12 mimics the increase in anxiety induced by prenatal HFD exposure, an effect correlated with maternal obesity and HFD exposure (Bilbo and Tsang, 2010; Sullivan et al., 2010) and possibly attributed to the increase in ENK levels in the PVN. Together with the evidence that a HFD increases circulating CXCL12 levels in the dam and the expression of CXCL12, CXCR4, and CXCR7 specifically in the PVN of the offspring, these findings indicate that CXCL12 may have a role in the behavioral effects of the HFD and focus attention on the PVN, a central structure involved in the integration of the behavioral responses. While measurements of the stress-related peptide CRF, which is densely expressed in the PVN, failed to reveal any change in response to CXCL12, a possible involvement of ENK together with this chemokine in the behavioral effects of prenatal HFD exposure is supported by the findings that CXCR4 receptors and ENK both exist in high concentrations in the PVN and are strongly stimulated by a HFD (Poon et al., 2016) and that ENK itself which was stimulated by CXCL12 is known to be a key component in stress responses (Konig et al., 1996; Bilkei-Gorzo et al., 2004, 2008; Melo et al., 2014). Whereas, chronic inflammation or HFD intake is shown to increase caloric intake and contribute to later-life obesity in adult animals (Dahlgren et al., 2001; Williams et al., 2011), these changes were not evident here in P50 offspring given short-term access to the HFD for only 7 days. This shows that the similar changes in emotional behavior induced by prenatal exposure to a HFD or CXCL12 can occur in the absence of changes in consummatory behavior or body weight.

### Prenatal CXCL12 mimics certain aspects of prenatal HFD exposure in offspring

A major question in the field of ingestive behavior is how the immune system and the consumption of a HFD during gestation affect the developing brain and the physiological and neural systems in offspring. As described above, the immune system, consisting of hundreds of cytokines and chemokines (Cameron and Kelvin, 2003), is markedly affected by HFD exposure in both adult and embryonic animals (Bilbo and Tsang, 2010; Reynolds et al., 2014), and it in turn affects the development of neuronal systems in the brain as well as multiple other organs (Boulanger, 2009; Deverman and Patterson, 2009). The current findings, in addition to providing further support for this, focus attention on a specific inflammatory mediator, CXCL12, and its role in altering a particular subset of peptide neurons and specific emotional behaviors in the offspring. With full or conditional knockout of this system found to be lethal or to cause severely abnormal development (Nagasawa et al., 1996; Ma et al., 1998; Mithal et al., 2012), further manipulations of this chemokine system to understand its essential role in mediating the effects of prenatal HFD exposure, and perhaps explain why the PVN is selectively activated by the CXCL12 system and the PFLH is differentially responsive, must await the commercial availability of effective antagonists and antibodies for *in vivo* use against CXCL12 or its receptors. While other chemokines such as CCL2 may be involved and have also been shown to stimulate the neuropeptides highly responsive to a HFD (Poon et al., 2014; Segovia et al., 2014; Chang et al., 2016), the function of these chemokines clearly differs from that of CXCL12 as they are not necessary for survival of the offspring (Kuziel et al., 1997; Lu et al., 1998). Together, the results reported here, showing a HFD to elevate circulating CXCL12 in the dam and increase levels of CXCL12 and its receptors in the PVN of offspring and also revealing similar effects of the HFD and CXCL12 on neurogenesis, neuropeptides, and behavior mediated by these brain systems, are consistent with the possibility that this chemokine, CXCL12, is involved in mediating some effects of prenatal HFD exposure.

## Author contributions

KP conceived research, conducted experiments, analyzed data, drafted manuscript, and approved submission. JB conducted experiments, revised manuscript, and approved submission. HS conducted experiments. GC conducted experiments. SL revised manuscript and approved submission.

### Conflict of interest statement

The authors declare that the research was conducted in the absence of any commercial or financial relationships that could be construed as a potential conflict of interest.
